# Review and Integrated Framework for Deep‐Sea Mineral Resources, Ecological Influence, and Future Development

**DOI:** 10.1155/ijm/9695812

**Published:** 2026-03-04

**Authors:** Jia Liu, Can Wang, Ini-Ibehe Nabuk Etim, Ruiyong Zhang, Wolfgang Sand, Xiao Wang, Luhua Yang, Yanchen Ge, Jiazhi Liu

**Affiliations:** ^1^ The School of Bioengineering, Qilu University of Technology (Shandong Academy of Sciences), Jinan, China, qlu.edu.cn; ^2^ State Key Laboratory of Advanced Marine Materials, Key Laboratory of Marine Environmental Corrosion and Bio-Fouling, Institute of Oceanology, Chinese Academy of Sciences, Qingdao, China, cas.cn; ^3^ University of Chinese Academy of Science, Beijing, China, ucas.ac.cn; ^4^ Marine Chemistry and Corrosion Research Group, Department of Marine Science, Akwa Ibom State University, Uyo, Nigeria, aksu.edu.ng; ^5^ Guangxi Key Laboratory of Marine Environmental Science, Institute of Marine Corrosion Protection, Guangxi Academy of Sciences, Nanning, China, gxas.cn; ^6^ Biofilm Centre, University of Duisburg-Essen, Essen, Germany, uni-due.de

**Keywords:** deep biosphere, deep-sea ecosystem, deep-sea mineral, deep-sea mining, marine bioleaching

## Abstract

As global industrialization advances and land‐based resources decline, deep‐sea resource development is becoming increasingly vital. The deep sea is rich in minerals such as polymetallic nodules, polymetallic sulfides, and cobalt‐rich crust. However, deep‐sea ecosystems and geochemical cycles can be severely impacted by extensive mineral mining. The extraction, collection, hoisting, washing, offshore processing, and transportation of minerals will impact the seabed, the water column, and the surface environment of the original marine area to varying degrees. Deep‐sea ecosystems are particularly fragile, with scarce sources of matter and energy, making the impacts of deep‐sea mining far more severe than anticipated until now. A comprehensive assessment of the ecological impacts associated with deep‐sea mineral extraction is essential to ensure a sustainable and responsible development of deep‐sea resources. Quantitative assessments indicate that sediment plumes generated by mining can disperse over 5–10 km from the operation site, while benthic community recovery may require several decades to centuries, underscoring the long‐term ecological risks involved. These assessments lay the basis for effective systems and standards for sustainable mining, environmental monitoring, and governance. This review summarizes the current state of deep‐sea mineral extraction and utilization, analyzes the ecological and environmental consequences of mining activities, and discusses emerging technologies and innovative approaches for recovering resources in the deep ocean. This study is aimed at serving as a scientific reference for researchers and policymakers, contributing to the development of international frameworks and standards for the sustainable exploitation of deep‐sea minerals.

## 1. Introduction

To address the increasing global demand for mineral resources driven by population growth and economic development—particularly in emerging economies such as China and India—there is an urgent need to develop innovative technologies for mineral extraction and utilization [1–9]. Conventional terrestrial mining has led to a range of economic, environmental, and social challenges, including the cost of mineral production, habitat degradation, air and water pollution, toxic waste generation, and the displacement of local communities [10, 11]. As easily accessible land‐based mineral reserves become progressively depleted, signs of supply fatigue are emerging. Consequently, attention is shifting toward the exploration and exploitation of marine mineral resources as a promising alternative to meet future resource demands [12, 13].

Deep‐sea mineral resources—such as polymetallic nodules (PMNs), polymetallic sulfides, and cobalt (Co)‐rich crusts—are rich in critical elements such as copper (Cu), Co, nickel, and rare earth elements (REEs) [14–16]. For instance, total deep‐sea reserves of manganese (Mn) crusts are estimated at 3 trillion t, with a recoverable Mn potential of approximately 75 billion t—over 40 times the Mn reserves on land. In the Pacific Ocean seabed, Co reserves reach about 50 million t, seven times the amount on land. Energy resources in the deep‐sea ground include oil, gas, and gas hydrates. Over the past decade, newly discovered offshore oil and gas reserves have constituted 60% of the world′s total reserves, with 62% of these located in deep and ultradeep waters. Gas hydrates make up 98% of the world′s marine reserves [17]. This abundance positions the deep‐sea domain as a critical and growing hub for global oil and gas exploration. These minerals are fundamental for modern applications spanning electronics, renewable energy systems, and advanced medical technologies [18].

Efficient mineral recovery is the most critical step in developing deep‐sea resources. At present, mainstream mining practices primarily rely on hydraulic or mechanical collectors to gather mineral ores, which are then transported to the surface of support vessels via pipeline systems for preliminary processing. However, operating within the extreme conditions of the deep sea—characterized an immense hydrostatic pressure and a complex seabed environment—poses formidable engineering challenges for mining equipment. Ensuring long‐term stability and reliability under such conditions remains a major technological bottleneck. For instance, transport pipelines are prone to clogging, while current lifting efficiencies are inadequate to support large‐scale commercial exploitation. Beyond engineering constraints, environmental concerns are equally pressing. Mining operations inevitably generate sediment plumes, which can disperse over several kilometers and may cause severe, long‐lasting damage to benthic ecosystems. Alarmingly, the recovery of these disturbed environments may require decades or even centuries, underscoring the ecological risks associated with current deep‐sea mining approaches.

**Figure 1:**
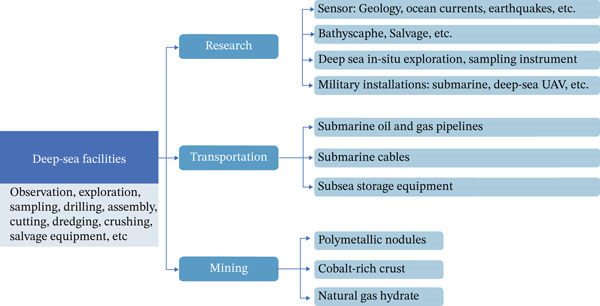
Primary types of deep‐sea engineering facilities adapted from [27, 28].

With increasing exploration in the deep sea, a variety of in situ sampling, detection, analysis, and transportation equipment has been deployed (Table 1). The US Army′s “21st Century Sea Power Cooperation Strategy” introduced in 2015 clearly points out that deep‐sea warfare is a vital matter of sea power gain or loss [24]. Addressing the corrosion of these facilities is a central focus to ensure their longevity and performance in extreme marine environments. Nowadays, deep‐sea facilities can be mainly divided into exploration, transportation, and mining. With the development of deep‐sea exploration, these facilities are already in a stage of rapid development [25, 26].To support an efficient exploration and green development of deep‐sea resources, a system of relevant deep‐sea engineering facilities has been gradually constructed and continuously improved. According to their functions, these facilities can be divided into three major types: scientific research, transport, and mining, as shown in Figure 1 [27]. Among them, scientific research facilities are mainly used for observation, sampling, and in situ experiments in the deep‐sea environment, which provide the necessary technical support (e.g., microbial diversity research and exploration of biological leaching mechanisms) [29]. For example, various types of sensors can achieve continuous monitoring of geological activities, ocean currents, earthquakes, and other parameters; submersibles and salvage devices are widely used for obtaining samples from deep‐sea sediments and minerals. In addition, the development of deep‐sea in situ detection and sampling equipment has made it possible to carry out observations of biological interactions in extremely high‐pressure and low‐temperature environments (always +4°C). Transport facilities include submarine oil and gas pipelines, deep‐sea communication cables, and submarine storage devices, which support the transfer of resources and information during deep‐sea development. Mining facilities directly serve the acquisition of important mineral resources such as PMNs, polymetallic sulfides, and Co‐rich crust, which are the core application scenarios of bioleaching technology [30]. The synergistic operation of these facilities not only enhances the efficiency of resource recovery but also provides a solid foundation for the exploration of low‐impact and sustainable deep‐sea microbial mining technologies [31].

**Table 1:** Progress in the development of facilities for exploiting deep‐sea mineral resources for the period, 2017–2024 [19–23].

Year	Country/company	Depth/m	Content
2017	Japan/Japan Oil, Gas and Metals National Corporation (JOGMEC)	1600	Mining vehicle collection and hydraulic lift test
2017	EU/Viable Alternative Mine Operating System (VAMOS)		Mining vehicle positioning, navigation, and sensing test
2017	Belgium/Global Sea Mineral Resources (GSR)	4571	Mining vehicle walking test
2018	Belgium, Germany/GSR, Federal Institute for Geosciences and Natural Resources (BGR)		Mining vehicle walking, nodule collection test, and environmental impact assessment study
2018	China/Changsha Research Institute of Mining and Metallurgy Co., Ltd.	514	“KUNLONG 500” mining vehicle sea trial
2018	China/Changsha Research Institute of Mining Co., Ltd.	2000	“KUNLONG 2000” cobalt‐rich crust large‐scale sampler sea trial
2019	Belgium/GSR	4500	Tracked walking and hydraulic collection test
2019	China/Changsha Research Institute of Mining Co., Ltd.	2900	Comprehensive sea trial of acoustic thickness measurement, walking, cutting, and collection
2019	China/Institute of Deep‐Sea Science and Engineering, Chinese Academy of Sciences	2498	Mining vehicle solo sea trial
2020	Japan/JOGMEC	1600	Cobalt‐rich crust trial mining
2021	Belgium/GSR	4500	Tracked walking, hydraulic collection, and environmental monitoring test
2021	India/National Institute of Ocean Technology (NIOT)	5270	Mining machine mobility and maneuverability test
2021	China/Dalian University of Technology	500	“Changyuan” intelligent mixed transport equipment sea trial
2021	China/Shanghai Jiao Tong University	1300	“Kaituo No. 1” deep‐sea heavy‐duty mining vehicle sea trial
2021	China/China Ocean Mineral Resources Research and Development Association	1306	Full series of linked deep‐sea mining tests
2022	China/Changsha Institute of Mining Research Cobalt, Ltd.	3500	Deep‐sea mineral mining trials
2022	Canada/The Metals Company (TMC)	4400	Polymetallic nodule collection, transport, and surface system test
2023	China/Changsha Institute of Mining Research Cobalt, Ltd.	> 1000 m	“Manatee II” deep‐sea heavy‐duty mining vehicle
2024	China/Changsha Institute of Mining Research Cobalt, Ltd.	6000	Intelligent electric drive deep‐sea heavy‐duty mining vehicle platform
2024	China/Shanghai Jiao Tong University	4000	“Kaituo No. 2” deep‐sea mineral mining trials
2024	China/Laoshan Laboratory	4000	Deep‐sea detection

**Figure 2:**
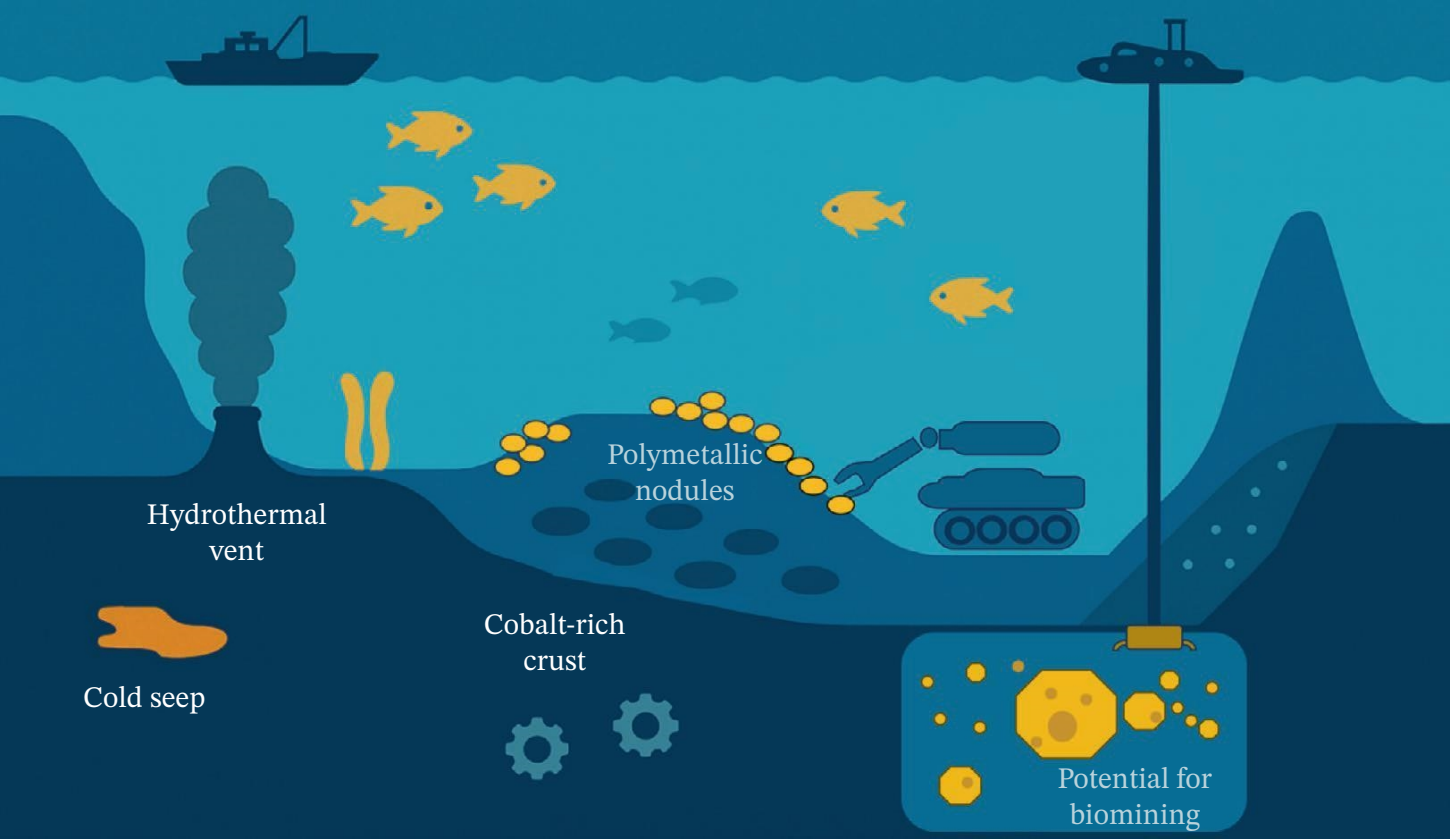
Development and utilization of deep‐sea mineral resources [33–36]. The diagram illustrates major deep‐sea mineral resources, including polymetallic nodules on the abyssal plain, cobalt‐rich crust on a seamount, and deposits near hydrothermal vents and cold seeps. A seafloor mining vehicle is shown collecting the nodules, which are then transported to a subsea facility.

Deep‐sea mining equipment is considered to be the largest deep‐sea operation system currently manipulable by mankind, covering industrial chain processes such as exploration, mining, metallurgical processing, and transportation. It integrates a full range of platforms and systems and equipment systems for seabed operation, underwater transportation, dynamic transmission and distribution, centralized control, and surface support [7, 8, 32]. As illustrated in Figure 2, the exploitation of these resources typically relies on the coordinated operation of marine vessels, underwater vehicles, and mining equipment. Deep‐sea mineral mining involves the following steps: First, the seabed mining system (e.g., auxiliary crushing machinery, excavation machinery, and mineral collection machinery) excavates, crushes, and collects minerals from the mineral deposit layer. Then, the slurry is transported via a seabed hose to an underwater lifting pump near the seabed. Subsequently, the slurry flows through a water separator system connected to the underwater lifting pump and is then sent to the dewatering system. Finally, after multistage dewatering and separation of the slurry, the minerals are transferred to the storage and transportation system [37]. After the slurry is separated by multistage dewatering, the minerals will be transported to land by barge; remaining seawater, marine microorganisms, and ultrafine slag will be pumped back into the seabed after pooling to minimize the damage to the marine environment. Currently, there is no commercialized deep‐sea mining system in the world. Most of the equipment is still in the development and testing stage [38–40].

However, the conventional approach of transporting deep‐sea minerals to land‐based facilities for processing entails significant challenges: It consumes vast transportation resources, increases costs, and aggravates environmental pollution in the deep sea. Moreover, the scarcity of essential leaching agents, such as freshwater, further restricts sustainable development. In response, increasing attention has been directed toward the application of marine microorganisms for in situ seawater bioleaching [33, 34]. This emerging technology leverages the unique metabolic capacities of deep‐sea microbes to extract valuable metals directly under marine conditions, offering a pathway to mitigate the ecological footprint associated with traditional mining methods. Nonetheless, the inherently complex composition of deep‐sea minerals poses serious limitations to the efficiency of conventional leaching strategies, including existing microbial approaches. Consequently, there is an urgent need to discover novel leaching microorganisms with enhanced adaptability and catalytic capacity, tailored specifically for the bioleaching of deep‐sea mineral resources.

## 2. Deep‐Sea Mineral Resources

The oceans cover about 71% of the Earth′s surface, and the deep sea, as a part of the oceans, is rich in mineral resources and has become a new frontier in the development of global mineral resources. With the gradual depletion of land‐based mineral resources, deep‐sea mining, as a potential direction of resource development, has gradually gained a high degree of attention from various countries. Therefore, an in‐depth understanding of the types of deep‐sea deposits and their geographical distribution is particularly important [35, 36, 41]. The basic types of seafloor marine deposits include PMNs, polymetallic sulfides, and Co‐rich crust. These deposits differ significantly in terms of their physical and chemical properties, formation conditions, metal content, geographic distribution, mining techniques, and environmental and social impacts.

**Figure 3:**
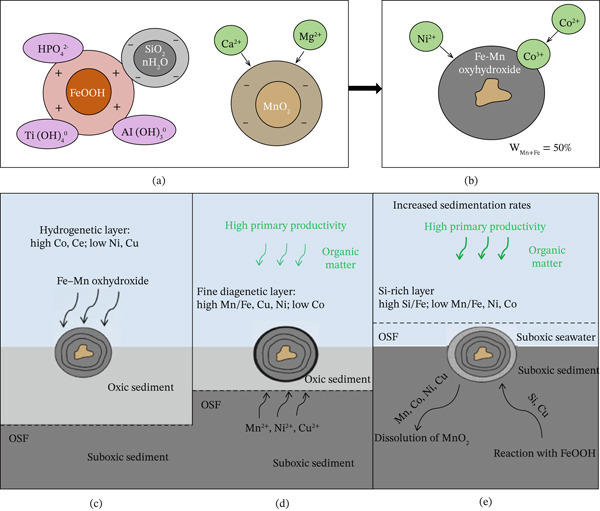
Illustration of the structure and formation process of polymetallic nodules, adapted from [33]. (a, b) Two stages of the formation of hydrogenetic layers. (c–e) The schematic of formations of hydrogenetic, diagenetic, and Si‐rich layers, with variations in chemistry. OSF = oxic–suboxic front. (a) OSF is deep, and the metals in pore water within suboxic sediments cannot diffuse to the bottom of the ferromanganese nodule to be adsorbed, which mainly experience oxic hydrogenetic processes. (b) OSF is near the bottom of the ferromanganese nodule, and the metals of pore water within suboxic sediment can diffuse upward, approaching the ferromanganese nodule. (c) OSF is above the ferromanganese nodule, and dissolution of nodules occurs.

PMNs, mainly composed of Mn, iron, nickel, Cu, and other metallic elements, are usually deposited in the form of nodules or clusters. They are widely distributed in areas such as the central and western parts of the Pacific Ocean and the abyssal plains of the Indian Ocean [42–44]. According to estimates, the total reserves of Mn nodules worldwide are about 300 billion t, of which the reserves in the Pacific Ocean amount to 170 billion t [42, 45]. Mn and iron oxides in Mn nodules are found in low concentrations in seawater, but Mn and iron usually dominate in these deposits, accounting for about 30% or more of the total mass. The formation of PMNs involves complex hydrogenetic and diagenetic processes, as illustrated in Figure 3 [46]. The hydrogenetic layer is characterized by high Co and Ce concentrations but low Ni and Cu, formed by the direct precipitation of Fe–Mn oxyhydroxides from seawater under oxidizing conditions. The formation of Mn nodules is a long process, usually requiring millions of years of accumulation, and their mineral composition and structure are influenced by currents, sedimentation rates, and other factors [43]. Seafloor polymetallic sulfides are formed by the deposition of sulfide minerals and are mainly enriched in Cu, gold, zinc, silver, and other metallic elements. They are usually found in areas of hydrothermal activity on the seafloor, such as mid‐ocean ridges, island arcs, and fracture zones in spreading basins. The formation of massive seafloor sulfide deposits depends on the activity of seafloor hydrothermal vents, and the cooling temperature and mineral‐rich environment provide conditions for the precipitation of metallic elements. Seafloor polymetallic sulfides contain high levels of Cu and gold. Their economic value is more prominent [44, 47]. Co‐rich crusts are similar to PMNs, since they consist mainly of Mn and iron oxides. But the environments, in which they are formed, are usually more specific, such as the surfaces of seamounts or on deep‐sea ridges [18, 48, 49]. The main characteristic of Co‐rich crusts is their high metal contents, especially when formed in deep‐sea waters. Thus, they have a high potential for commercial exploitation. Co‐rich crusts contain higher metal concentrations than Mn nodules and tend to be enriched in elements such as Co and nickel.

The distribution of different types of deep‐sea deposits varies globally (Table 2). PMNs are concentrated in the central and western Pacific Ocean, particularly in the Clarion–Clipperton zone (CCZ) of the eastern Pacific Ocean. There are huge reserves of Mn nodules, estimated to be up to 21 × 10^9^and 6 × 10^9^ t of Mn [48]. Co‐rich crusts, on the other hand, are found in the Pacific Ocean near the equator, particularly in the international seabed area and in the exclusive economic zones of some countries [66]. In addition, massive seafloor sulfides are mainly found on mid‐oceanic ridges and in hydrothermally active areas, especially on the East Pacific Rise and the Mid‐Atlantic Ridge, among other areas [49, 67]. Despite the promising prospects for the development of deep‐sea mineral resources, the exploitation of these resources may have a serious impact on the deep‐sea ecosystem, especially as the exploitation of deep‐sea deposits may damage the seabed ecosystem and affect biodiversity. Therefore, the sustainable development of deep‐sea mineral resources and environmental protection have become important issues in the development of global mineral resources [51, 52].

**Table 2:** Deep‐sea rare ore distribution.

Mineral	Distribution Areas
Polymetallic sulfides	• East Pacific Rise (EPR): 13°–21°N section, dense black smoker vents, rich in Cu, Zn, and Au [46]
	• Mid‐Atlantic Ridge (MAR): TAG hydrothermal zone, sulfide Co content up to 0.5%
	• Southwest Indian Ridge (SWIR): Longqi hydrothermal field, massive and chimney‐like sulfides
Polymetallic nodules (Mn–Fe)	• Clarion–Clipperton zone (CCZ): Central Pacific, 4000–6500 m depth, ~6 million km^2^ coverage, resource estimate 2–3 trillion t [50]
	• Peru Basin: South Pacific, manganese content up to 25%, enriched in Co and nickel [51]
	• Central Indian Ocean Basin: Diagenetic nodules with high Cu and nickel [52]
Cobalt‐rich crusts	• West Pacific Magellan Seamounts: 800–2500 m depth, Co content up to 1.7%, total REEs > 1000 ppm [53]
	• Central Pacific Line Islands Seamounts: OMZ depth 400–1000 m; crusts grow on seamounts/ridges (slope < 15^°^), mainly at 1500–2500 m
	• Atlantic Seamounts (Brazil Margin): Crusts with Te content up to 50 ppm
Deep‐sea REE Sediments	• Eastern South Pacific: 4000–5000 m depth, REE content up to 8000 ppm, MREE/HREE > 50*%* [54]
	• North Central Pacific (CCZ south): REEs in biogenic apatite
	• SW Pacific Manus Basin: REE–zeolite associations with high extraction potential [55]
Phosphorites	• East Pacific Margin (Peru–Chile Trench): P_2_O_5_ content up to 20%
	• Mid‐Atlantic Romanche Fracture Zone: Hydrothermal‐related phosphorites [56]
	• Indian Ocean Crozet Seamounts: Upwelling‐related phosphorites
Zeolite deposits	• East Mariana Basin (West Pacific): 6100 m depth, zeolite–apatite associations [57]
	• Macquarie Ridge (South Pacific): Volcaniclastic sediments [58]
	• Iceland Ridge (North Atlantic): Clinoptilolite formed via hydrothermal activity [59]
Barite deposits	• EPR hydrothermal vents: Barite–sulfide associations.
	• MAR TAG hydrothermal zone: Barite veins with Ba content up to 65% [59]
	• Carlsberg Ridge (Indian Ocean): Platy barite on sulfide mounds
Ilmenite sands	• Blake Plateau (North Atlantic): 4000 m depth, magnetite–nodule associations
	• Coral Sea (South Pacific): Slope sands with 45% magnetite [60]
	• Arabian Sea (Indian Ocean): Turbidite deposits with magnetite–quartz mixtures
Monazite sands	• Magellan Seamounts (West Pacific): 2000–3000 m depth, monazite–zircon associations
	• West African Margin (South Atlantic): Slope sands with REEs > 1000 ppm [61]
	• Chagos Ridge (Indian Ocean): Volcanic rock weathering products with granular monazite [62]
Cu–Ni sulfides	• Mariana Trough (West Pacific): Back‐arc spreading center, Cu content up to 5% [63]
	• EPR 13°N: Nickel content > 2*%*.
	• SWIR Longqi field: Stockwork Cu–Ni sulfides
Barium ferromanganese deposits	• EPR vents: Barite–barium ferromanganese associations
	• MAR TAG Zone: Colloform barium ferromanganese in sulfide pores [64]
	• SWIR Longqi field: Low‐temperature fluid‐derived deposits [65]

Abbreviations: EPR, East Pacific Rise; MAR, Mid‐Atlantic Ridge; MREE/HREE, middle/heavy rare earth elements; OMZ, oxygen minimum zone; REE, rare earth element; SWIR, Southwest Indian Ridge.

## 3. Deep‐Sea Mining and Mineral Processing and Their Influences

### 3.1. Deep‐Sea Mining

Deep‐sea mineral resource development is considered one of the largest and most complex marine engineering systems. It encompasses the full industrial chain from exploration and mining to metallurgy and transportation. This system integrates comprehensive operational platforms for seabed excavation, subsea transportation, dynamic power transmission, centralized control, and surface support [68]. A typical deep‐sea mining system consists of a seabed mining vehicle, a lifting and transportation system, a slurry separation and storage subsystem, and a surface support vessel. The primary objective is to achieve a stable and efficient extraction of PMNs, Co‐rich crusts, and seafloor massive sulfides (SMSs) under extreme conditions of high pressure and low temperature due to depths of several thousand meters [69].

**Figure 4:**
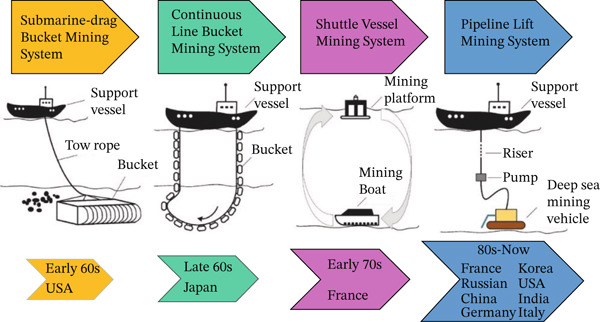
Development of deep‐sea mining systems [70].

At present, global deep‐sea mining technologies can be categorized into four representative system types (Figure 4): the dragline bucket mining system, the continuous line bucket mining system, the ocean shuttle mining system, and the pipeline lifting mining system. The dragline bucket system employs a mother ship to tow a cable‐driven mining bucket for ore collection. Although structurally simple, it is difficult to operate, inefficient, and unable to maintain a fixed mining trajectory. The continuous line bucket system uses a chain of buckets for continuous ore collection. It is easy to manufacture and cost‐effective but prone to entanglement accidents that compromise operational safety and productivity. The ocean shuttle mining system relies on autonomous underwater shuttles for transporting mined materials. It provides high automation and operational flexibility but is constrained by the endurance of onboard batteries and high manufacturing costs. The pipeline lifting mining system, by contrast, uses a fixed riser and hydraulic pumps to continuously transport slurry from the seafloor to the surface vessel. Due to its advantages of continuous operation, a high mining efficiency, and relatively low energy consumption that are achieved, it is widely recognized as the most promising technology for future commercial deep‐sea mining operations.

The mining equipment that enables these systems comprises several key components. The seabed mining vehicle is responsible for cutting, crushing, and collecting mineral deposits. Typical designs include hydraulically driven cutters and crushers mounted on tracked mining vehicles, as well as hydraulic suction and mechanical collectors. Additionally, navigation systems are based on optical fiber communication and acoustic positioning. For instance, the British company Soil Machine Dynamics (SMD) developed the Bulk Cutter and Auxiliary Cutter for Nautilus Minerals, which have undergone multiple field trials in polymetallic sulfide areas. The lifting system serves as the vital link between the seafloor and the surface, transporting the slurry through hydraulic riser pipes powered by subsea booster pumps, typically multistage impeller or diaphragm pumps. The surface support vessel provides energy, storage, separation, and data control functions, equipped with a dynamic positioning system (DPS) to maintain stability in rough sea conditions. Notable international projects include Japan′s Deep Sea Mineral Resources Explorer (DSMRE), which has achieved continuous mining trials at depths of 1600 m; South Korea′s MineRo II, which completed a successful 1300‐m sea trial; and China′s independently developed prototype systems based on the Jiaolong and Haiyi platforms, which have been tested in the PMN fields of the Western Pacific. Overall, deep‐sea mining equipment is evolving toward modularization, automation, and intelligent operation, significantly reducing human intervention and enhancing real‐time monitoring and control capabilities.

Nevertheless, the potential ecological impacts of deep‐sea mining on marine ecosystems have raised serious global concerns. Deep‐sea ecosystems are characterized by low energy input, high biodiversity, and extremely slow recovery rates, making them highly vulnerable to anthropogenic disturbances. Mining vehicles and cutting tools generate substantial benthic disturbance and suspended sediment plumes, which can spread over several kilometers, smothering benthic organisms, impeding filter feeders, and causing large‐scale habitat degradation. Studies have shown that ecosystem recovery in disturbed areas may take several decades. Additionally, mechanical noise from mining operations—often exceeding 180 dB—can interfere with the acoustic communication and migration patterns of deep‐sea fish and cetaceans, while intense artificial lighting can disrupt the circadian rhythms of phototactic species. During mineral crushing and slurry transport, heavy metals such as Cu, nickel, and Mn may be released into the surrounding seawater, posing toxic threats to benthic microbes and plankton. Some studies indicate that these metals can bioaccumulate through the food web, threatening higher trophic‐level organisms.

To mitigate such risks, countries are actively developing ecological compensation and environmental monitoring technologies, including the establishment of buffer zones around mining sites, deployment of environmental sensor networks (EMS), implementation of postmining ecological restoration experiments, and adoption of standardized environmental impact assessment (EIA) procedures under the framework of the International Seabed Authority (ISA).

In summary, advancements in deep‐sea mining technology have enabled humanity to explore and extract resources from several thousand meters below the ocean surface. Yet these capabilities bring profound challenges to the marine biosphere. Future development must prioritize the principles of “green mining” and “ecologically responsible design,” emphasizing energy efficiency, noise control, and sediment plume management. Strengthened international cooperation, transparent regulation, and rigorous long‐term ecological monitoring are essential to balance resource utilization and environmental conservation, ultimately promoting the sustainable development of deep‐sea mineral mining.

### 3.2. Deep‐Sea Mineral Leaching

Mineral leaching is the process of dissolving and extracting useful metals or other valuable constituents from ores by means of a chemical reaction between a specific solvent and the ore. The leaching processes for deep‐sea minerals, including PMNs, polymetallic sulfides, and Co‐rich crusts, largely rely on conventional terrestrial hydrometallurgical techniques, as no fully mature deep‐sea‐specific leaching technologies have been commercialized. For PMNs, ammoniacal leaching using ammonia–ammonium carbonate solutions and acid leaching with HCl or H_2_SO_4_ remain the most widely studied methods, with bioleaching emerging as an environmentally benign but comparably slow alternative. Polymetallic sulfides are typically treated through pressure oxidation (POX) leaching, bio‐oxidation leaching, or sulfuric acid leaching coupled with solvent extraction and electrowinning (SX–EW) for Cu and zinc recovery. Meanwhile, Co‐rich crusts are processed through acid or reducing acid leaching using agents such as SO_2_ or H_2_O_2_ to enhance the dissolution of Co and Ni and more recently through ionic liquid leaching systems designed to improve selectivity and metal recovery. However, despite the diversity of laboratory and pilot‐scale research, these leaching techniques remain adaptations of land‐based processing methods applied to simulated deep‐sea ore samples under controlled conditions. Real in situ or subsea hydrometallurgical extraction has to be realized due to challenges such as high hydrostatic pressure, corrosive extreme corrosion environments, reagent recovery, and ecological risk management. Consequently, deep‐sea mineral leaching remains an extension of conventional onshore metallurgical practices rather than a fundamentally new process under marine conditions, underscoring the need for technological breakthroughs tailored to subsea conditions.

### 3.3. Potential of Bioleaching for Deep‐Sea Minerals

Bioleaching is an innovative method for microbial extraction of metals and is recognized as a low‐carbon metal extraction technology [5, 71]. Microorganisms, through their life activities, oxidize or reduce the useful components of resources by their inherent oxidative and reductive properties. This process separates valuable metals from the original substance, releasing them in the form of ionic states or precipitates in aqueous solution or by relying on microbial metabolites to interact with minerals to dissolve and extract the useful components [72]. This method is based on the metabolism of microorganisms that produce various chemicals, such as organic acids and other oxidants, which promote the release of metals from solid ores by reacting with the minerals [73].

Compared to traditional metallurgical methods, bioleaching is gentler in reaction conditions and less intrusive to the environment. It consumes significantly less energy because it does not require extensive high‐temperature processing, and it does not produce large amounts of harmful chemicals during metal extraction [40]. Deep‐sea bioleaching refers to the prospect of employing specialized deep‐sea microbe species to purify metallic elements from deep‐sea minerals, likely in controlled processing facilities, rather than in situ on the deep seafloor. However, the mineral composition of deep‐sea minerals makes it difficult for traditional bioleaching strains to achieve efficient extraction. The development of new deep‐sea leaching microorganisms is necessary for the extraction of minerals from deep‐sea deposits. Currently, the research and development of bioleaching processes specifically leveraging deep‐sea microorganisms are in the primary period, with limited studies. Deep‐sea microorganisms, having evolved under high pressure, low temperature, and unique geochemical conditions, possess distinct metabolic pathways and enzyme systems [74]. These properties might confer advantages in dissolving complex deep‐sea minerals that are recalcitrant to conventional bioleaching, potentially leading to increased metal recovery rates and reduced processing costs and energy consumption [75].

Microorganisms relevant to bioleaching can include both autotrophic and heterotrophic organisms. Autotrophic microorganisms use sulfide, nitrates, ammonium salts, hydrogen gas, and Fe (II) as an energy source to oxidize and leach metals from minerals through oxidative reactions; heterotrophic microorganisms rely mainly on the secretion of organic acids to lower the pH around the ore, thus achieving the purpose of dissolving minerals [76]. In the specific leaching process, the understanding of bioleaching is less than that of chemical leaching, which may be due to the fact that bioleaching requires too many parameters to be adjusted [77, 78]. In addition, the leaching effect needs to consider the deterioration of microorganisms in addition to solids dissolution. Microbial facilitation of the leaching process includes both direct and indirect contact mechanisms. The direct contact mechanism involves the leaching microorganisms coming into contact with the mineral surface, whereby mineral decomposition occurs through direct oxidation of the minerals and energy is gained through the action of enzymes, as well as dissolution of the mineral lattice [79–81]. In the copresence of water and air, the direct contact leaching reaction of metal sulfide ores occurs by mixed cultures or terrestrial archaea bacteria such as iron‐oxidizing bacteria (IOB) (i.e., *Acidithiobacillus ferrooxidans*, *Acidimicrobium ferrooxidans*, *Ferroplasma acidarmanus*, *Sulfolobus yangmingensis*, *Metallosphaera prunae*, *Acidianus manzaensis*, and *Acidianus sulfidivorans*).

**Figure 5:**
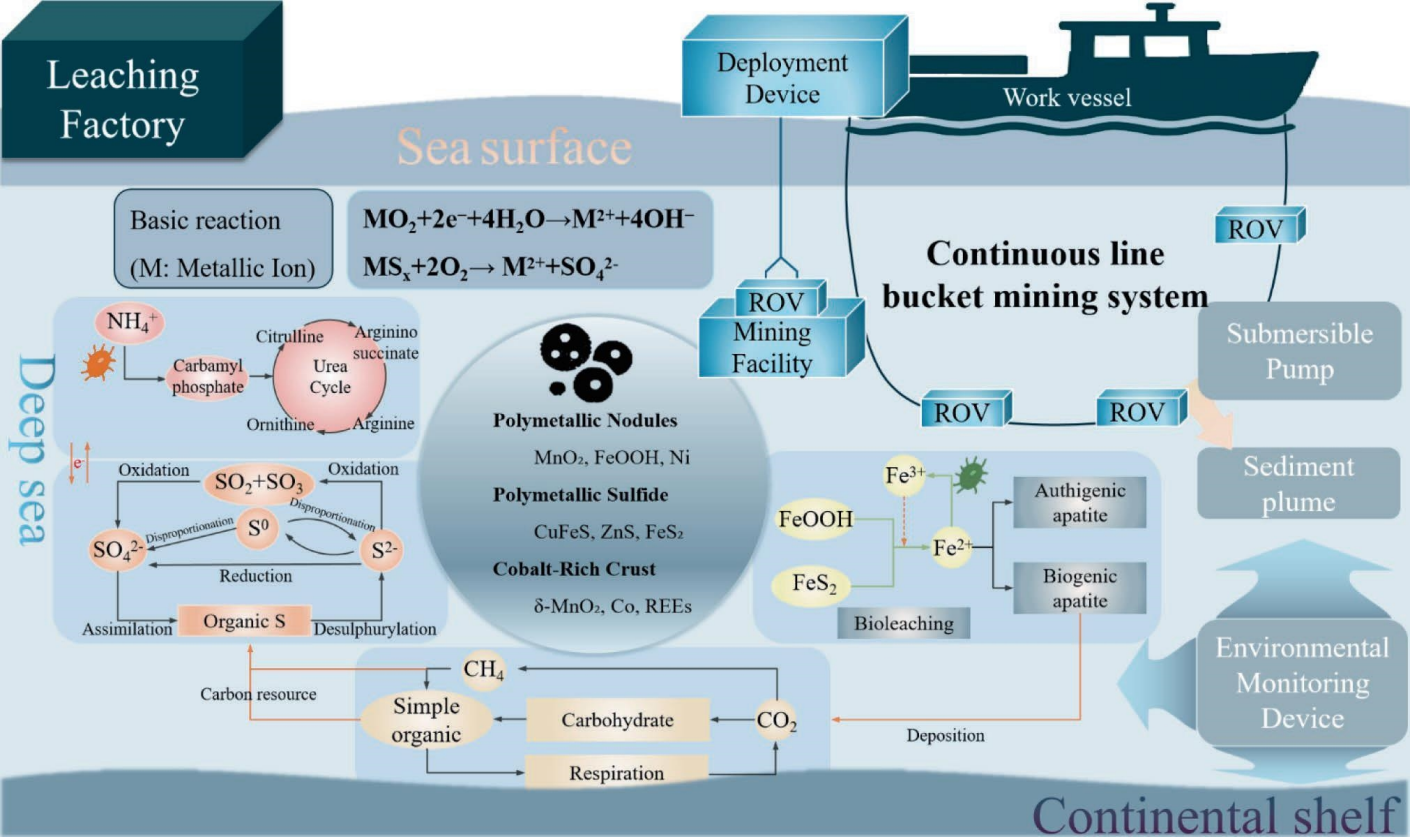
Schematic representation of microbial‐driven metal–mineral cycling and bioleaching processes in deep‐sea environments [72, 82–84]. The diagram illustrates an active mining system (top) targeting deep‐sea minerals (nodules, sulfides, and crusts). Below, it details the interconnected microbial‐driven sulfur, carbon, and urea cycles, which are responsible for natural bioleaching and the transformation of metal‐bearing minerals in the deep‐sea environment.

To ensure both metal recovery efficiency and ecological sustainability, it is essential to integrate bioleaching processes with adaptive engineering systems and real‐time geochemical monitoring. Such integration not only enhances the controllability of microbial activity under extreme conditions but also provides a framework for minimizing ecological disturbances. Figure 5 presents an integrated schematic model, which illustrates the key biochemical, geochemical, and engineering processes involved in deep‐sea microbially mediated mineral cycling and bioleaching. This system highlights how microbial metabolic activities interact with redox reactions of metal‐bearing minerals and how these processes are embedded within a larger operational framework involving remote mining technology and environmental monitoring [85].

In the deep‐sea environment, microorganisms drive the transformation of sulfur species through a series of oxidation–reduction and disproportionation reactions. Sulfate ions (SO_4_^2−^) are reduced to sulfide ions (S^2−^) under anaerobic conditions by sulfate‐reducing prokaryotes, followed by oxidation and transformation into elemental sulfur (S^0^), thiosulfate (S_2_O_3_^2−^), or other intermediates. These redox processes facilitate the breakdown of polymetallic sulfides such as CuFeS, ZnS, and FeS_2_, releasing metal ions into the surrounding environment. Meanwhile, microbial desulfurization and assimilation of organic sulfur compounds further contribute to the mobilization of sulfur‐bound metals. Additionally, nitrogen and carbon metabolic pathways (e.g., urea cycle and methane respiration) provide essential energy and redox equivalents that support microbial survival and activity under high‐pressure, low‐temperature conditions [82]. These processes are especially important for sustaining microbial consortia responsible for metal solubilization in PMNs, Co‐rich crusts, and other mineral substrates (e.g., *δ*‐MnO_2_, FeOOH, nickel, Co, and REEs).

A key component of the bioleaching pathway is the microbial oxidation of iron‐bearing minerals. Microorganisms convert FeS_2_ and FeOOH into soluble Fe^2+^ and Fe^3+^ species, which then participate in secondary mineral formation such as authigenic and biogenic apatite. These biomineralization processes not only enhance metal availability but also influence the geochemical stability of the seafloor ecosystem. The two fundamental leaching reactions shown in the diagram represent the oxidation of metal oxides and sulfides, providing the thermodynamic basis for metal solubilization in microbial environments:

MOx+244e−+H2O⟶M2++OH−MSx+2O2⟶M2++SO42−

These interactions form a comprehensive and sustainable deep‐sea bioleaching model that couples biological transformation with physical extraction technologies.

The proposed concept of deep‐sea microbial leaching aims to leverage specific strains of autotrophic and heterotrophic bacteria, potentially cultivated and optimized for ex situ processing of deep‐sea minerals. The goal is to efficiently dissolve metals from these minerals through their unique metabolites, such as organic acids and or oxidative enzymes, which are active under conditions that can be challenging for terrestrial microbes [86, 87]. However, it is crucial to recognize that the deep‐sea mining operations will lead to numerous subsequent effects, including the potential suspended deposition of tailings, which can profoundly affect benthic microbial communities over long distances by impairing their ecological functions. A recovery is often slow, and it is difficult to fully restore key ecological conditions. Therefore, any future development in deep‐sea mineral processing, including advanced bioleaching concepts, must be integrated with robust environmental management strategies. The long‐term vision could involve a closed‐loop process of “Enrichment‐Leaching‐Restoration‐Assessment,” quantitatively monitoring trace metals, pH, redox potential, and bioindicators such as benthic foraminifera in pore water while recovering valuable metals [87]. Relying on the self‐healing network constructed by microbial biofilms and extracellular polymers, this model could not only reshape the disturbed sediment structure and promote the mineralization of sulfides but also provide continuous biogeochemical health monitoring and restoration strategies for the deep‐sea environment. Thus, it would promote the synergistic development of deep‐sea resource utilization and ecological protection [88, 89].

## 4. Impacts of Mining on the Deep‐Sea Environment

### 4.1. Impacts of Mining on Deep‐Sea Ecosystems

The environmental impact of deep‐sea mining has always been a great concern to the international community [86, 90]. Over the past 50 years, some countries and scientific organizations have investigated many environmental problems caused by deep‐sea mining. The development of technologies for monitoring, assessing, and restoring the environmental impacts of deep‐sea mining, as well as the achievement of a balance between deep‐sea mining and environmental protection, is an indispensable path to the commercial exploitation of deep‐sea mineral resources. The mining industry is considered to be the world′s largest generator of waste, and providing safe disposal sites for mining waste poses significant environmental challenges.

PMNs, massive sulfides, and Co‐rich crusts are important geological carriers that support mineral resources and provide irreplaceable ecosystems for deep‐sea biota. For example, although PMN reserves in the CCZ are 5–10 times greater than terrestrial resources, this equatorial Pacific “resource base” is a biodiversity hotspot with about 100 endemic macrofauna species within a 30 × 30 km area, as well as unique species not found in the sediment and water column, and unique microbial communities not found in sediments and water bodies. This high degree of overlap between resources and ecology presents a dual challenge for the development of mining technology [87, 91]. Polymetallic sulfides from active hydrothermal vents provide habitats for novel faunal communities, reshaping our understanding of primary energy sources and the origin of life. These habitats also exhibit significant structural and ecological variation at both local and regional scales. While polymetallic sulfide mining is primarily expected to target “extinct” hydrothermal vents—due to the highly corrosive nature of hot vent fluids—active vents remain unprotected under current regulations. In addition, from a biome perspective, extinct vents, although understudied, are still studied. Ferromanganese‐encrusted seamounts have been identified as hotspots that support biodiversity that varies within seamount chains. The principal ecological impacts of deep‐sea mining on benthic organisms include sediment redeposition—likely the most influential disturbance despite the lack of defined ecological thresholds—and heavy metal toxicity, which is further complicated by factors such as metal bioavailability. A field‐scale simulation of sediment disturbance during seafloor mining was conducted in the PMN province of the western Pacific Ocean at a depth of 5700 m using the Field Disturbance and Observation Unit (FDOU). It integrates multiple sensor systems for comprehensive environmental monitoring. According to the study, in the PMN zone, the impact of the stroking and uplifting processes on the bottom sediments is 30 times greater than that caused by waves or currents. The disturbance affects the bottom turbidity over a distance of about 126 m. The time required for the disturbance to return to normal is about 4 h, and the area of influence is about 1000 m^2^. At the same time, resuspension of bottom sediments leads to density anomalies and increased salinity. During the observation period, the phenomenon of deep‐sea reciprocating currents was observed, which may have led to the creation of a suspended sediment cloud in the waters near the bottom of the mine site as a result of the suspended sediment from the continuous operation of the mining vehicles. This could lead to a sustained increase in nutrients and a sustained decrease in dissolved oxygen in the waters near the bottom of the mine site, which would have a significant impact on the local ecosystem. Consequently, the manner in which mining vehicles are dredged and washed in the water column may have a significant impact on the marine environment. Suction beneficiation technology is recommended for mining vessels, and the separated sediments are brought back to land for integrated use [91].

While deep‐sea mining would certainly harm the environment, deep‐sea mining is still a better option than land‐based mining if environmental regulations are effectively enforced and compliance is monitored. This is primarily because deep‐sea mineral resources hold greater potential, their extraction causes less impact on the surface environment, and they can, to some extent, alleviate the conflict between the depletion of terrestrial mineral resources and their development. However, as the methods of land‐based mining and deep‐sea mining are very different, no direct comparison has been made between them [92]. Mineral deposits were extracted using the ecosystem approach to compare greenfield open‐pit deposits with deep‐sea mines in the Papua New Guinea Special Economic Zone. It was found that deep‐sea mining appeared to be a desirable alternative to land‐based mining and that deep‐sea mining appeared to be better suited than land‐based mining to all industries except energy and raw materials (e.g., food, pharmaceuticals, and water supply), as far as the provision of services was concerned. However, there are many issues that have been neglected in deep‐sea mining so far. The choice of deep‐sea mining must also be based on an environmental assessment to ensure that the environmental impacts are minimized. This will require more legislation and policy support to leave us with a deep‐sea environment that can be sustainably exploited for ourselves and future generations [93].

### 4.2. Ecological Remediation of Deep‐Sea Mineral Leaching

Deep‐sea ecosystems are the most extensive ecosystems on Earth. They provide key goods and services for human well‐being, such as genetic resources and climate regulation. The deep oceans are rich in mineral resources and are seen as key to meeting the growing global demand for new energy technologies [94]. Maintaining the sustainable functioning of the global biosphere, therefore, requires the protection of deep‐sea ecosystems, in particular as they face significant changes related to human and climate‐induced impacts. Deep‐sea mining is currently considered to be a than suitable method of accessing rare mineral resources. However, sediment fallout from deep‐sea mining can travel great distances and cause harm to benthic organisms. In addition, recovery of deep‐sea ecosystems is very slow, and some key ecological functions may not be fully restorable. The April 3, 2024, issue of PNAS notes that sediment dust from deep‐sea mining can spread over great distances and harm benthic organisms [33]. Furthermore, the recovery of deep‐sea ecosystems may be extremely slow, and some ecological functions may never fully recover. In the face of these potential ecological risks, scientists are calling for more in‐depth studies to be conducted before commercial mining takes place. One study found that the effects of a simulated deep‐sea mining experiment conducted in the Peru Basin in 1989 are still visible today. Now, 26 years later, the ecosystems in the affected areas have not yet fully recovered. There are reduced populations of suspended filter‐feeding animals and impacts on the carbon cycle [95]. Comprehensive, long‐term monitoring and assessment of the deep‐sea environment, including physical, chemical, and biological parameters, before, during, and after mining, are needed to understand the extent of ecosystem change and damage. Rigorous environmental testing and assessment are important factors in ensuring the stability of regional ecosystems. Measurements of the depth distribution of dissolved trace metals, pH, and redox potential in sediment pore waters are considered suitable for monitoring biogeochemical processes in sediments from areas affected by mine tailings. Benthic foraminifera are part of the meiofauna, which can be used to assess biological impacts. Because of their high sensitivity to environmental change, they have become good bioindicators for natural stress and anthropogenic disturbances. Although we lack data to assess the spatial scales of degraded deep‐sea habitats, many studies have documented human impacts throughout the oceans [95]. In order to address these impacts, the ISA has mandated that contractors conduct baseline investigations and EIAs as part of its Environmental Management Plan. In order to define environmental reference states, baseline data must include physical, chemical, and biological parameters. The physical parameters include sediment and currents, while the chemical parameters include trace metals, pH, and redox potential. The biological parameters include foraminifera and meiofauna. In the context of pilot mining tests, real‐time monitoring systems have been employed to track sediment plume behavior and habitat changes. These systems integrate benthic landers, turbidity and redox sensors, and ROV imaging to achieve this objective. In instances where these limits are surpassed, adaptive management actions, including adjustments to discharge locations, are initiated.

However, conservation measures alone will not be sufficient to reverse the trend of deep‐sea habitat degradation. Deep‐sea restoration actions may be scientifically feasible, but when applied at a broad scale, it remains questionable whether they will achieve sustainability goals [96]. Successful implementation of most restoration efforts will at first require a deeper understanding of biodiversity and deep‐sea ecosystem functioning, as well as a better understanding of ecosystem resilience and recovery rates of the deep‐sea fauna. In addition to limited data availability, expensive technology (estimated to cost millions of dollars) is a major barrier to large‐scale deep‐sea restoration, but international collaboration (e.g., increased collaboration between industrial and academic scientists) could significantly reduce this operational cost. Future deep‐sea ecosystem restoration may provide important business opportunities for technology development and applications and investment in natural capital for new and competitive blue growth industries [93].

### 4.3. Tailing Disposal and Environmental Protection Issues

Bitumen extracted from the Athabasca Oil Sands Region (AOSR) plays an important role in meeting global and North American energy needs [97–99]. However, tailings from the extraction and disposal processes in the area have given rise to a variety of environmental problems. For every cubic meter of oil extracted from the oil sands, approximately 4 m^3^ of tailing waste is generated and stored in a tailing storage facility on site [100, 101]. The total amount of flowing tailings in the AOSR is reported to be over 1.25 billion m^3^. Deep‐sea tailings are produced during the mining process and are disposed of in deep‐sea areas. Typically, this happens from seabed mineral extraction (e.g., mining of deep‐sea Mn nodules and hydrothermal sulfides) or from the discharge of mine tailings from land‐based mines into deep‐sea areas by transport such as pipelines [102]. The discharge of deep‐sea tailings likely has impacts on marine ecosystems, including siltation due to excess sediment formation, metal toxicity, chemical toxicity, changes in organic content, oxygen depletion (→anaerobic conditions favoring reductive processes→liberation of metal cations), particulate matter suspension and disturbance, and hydrodynamic changes. The discharge of tailings can lead to an increase in the concentration of particulate matter in the water body, affecting water quality. Based on environmental protection standards, the concentration of particulate matter in the water body should be controlled below 10 FTU (Formazin Turbidity Unit). And the thickness of the deposited tailings should be less than 10 cm in total, or the deposition rate should be less than 6 mm/year. This presents an innovative approach to comprehensively treat gold mine tailings [103]. The main focus is on the recovery of valuable feldspars and the safe landfill of the remaining low‐value components caused by a two‐step process. Firstly, magnetic separation and flotation are used to remove harmful iron impurities from the feldspar. Then, the deslimed gold tailings are flown in an acidic environment to recover the feldspar concentrate [102]. Finally, the ultrafine tailings, flotation tailings, and ferrous impurities are filled into the ground by cementation. This mixed‐use scheme aims to maximize the potential of the gold mine tailings for environmentally friendly disposal. In order to better manage the impacts of tailing discharges on the deep‐sea environment [104, 105], describe the development of an Environmental Adaptive Management System (EAMS) in contrast to management based solely on the quantity of discharges. The main driving forces for the development of a new management system for submarine tailings ponds were the desire to create a system based on what all stakeholders consider important (i.e., environmental impacts) [106]. In order to better manage the impact of tailing discharge on the ecological environment, continuous monitoring and targeted research are needed. Monitoring and research should be carried out on the water bodies, sediments, and organisms to understand the actual impact of tailing discharge. Management measures should be adjusted through the results of the monitoring in order to achieve an adaptive, optimized management. In conclusion, tailing discharge can have multiple impacts on the marine ecosystem. Comprehensive adaptive management measures are needed to minimize these impacts [107].

### 4.4. Effects of Sediment Plumes

**Figure 6:**
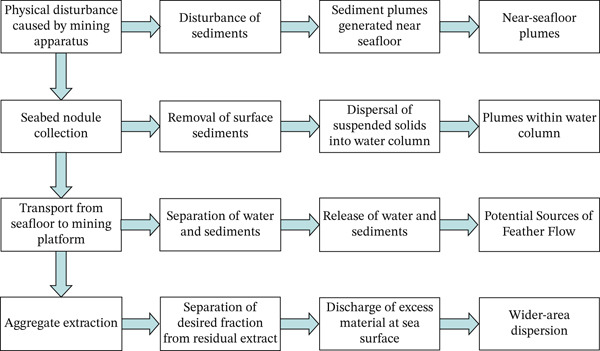
Schematic of plume formation processes associated with deep‐sea mining [108–110].

Sediment plumes refer to specialized water currents generated on the ocean floor during seabed disturbance or mineral extraction activities. They are characterized by the suspension of large quantities of particulate matter. During mineral extraction, sediment plumes undergo a complete cycle of disturbance→dispersion→sedimentation→resuspension (Figure 6) [108]. Primary sources of disturbances include mining equipment, concentrators, conveyor pipelines, water jetting, crushing/grinding, and excavation operations. These activities directly disturb seafloor sediments, mixing mineral particles, fine silt, organic matter, and pore water into the water column to form high‐concentration suspensions. The “near‐bed plume” formed near the seafloor typically remains confined within a few meters above the substrate, while some suspended particles may be transported into higher water layers, where they form a “midwater plume” or “turbidity plume.” Key impacts are summarized in Table 3.

**Table 3:** Main risks and impacts of deep‐sea polymetallic nodule mining [23, 111, 112].

Altered environmental conditions	Environmental impacts	Potential scope of impact	Duration of impact and potential for recovery
Sediment disturbance	Silt‐laden currents: Reduced light penetration, diminished phytoplankton growth, and cascading effects on productivity	An area of thousands of square kilometers	Affected during mining operations; once operations cease, recovery will be rapid
	Particle load: May cause suffocation of organisms	Depending on mining processes and local currents	Affected during mining activities; recovery is slow, particularly in severely impacted areas
	Sediment grain size alteration: Leading to changes in biological habitats	Sediment plume impact zone	The duration is influenced by changes in the sediment particle size distribution
Alteration of seabed topography	Thoroughly disturb the seabed and its compaction	Mining area	Long‐term (hundreds to thousands of years) impact; recovery is extremely slow
Habitat alteration	Changes in population density and structure	Mining area and plume impact zone	Recovery may be very slow
Noise impact	The masking effect on marine mammals	Low‐frequency noise can propagate 600 km	Once activity ceases, recovery is swift

The dispersion and transport of sediment plumes are governed by hydrodynamic conditions, particle characteristics, and convective stability. Bottom turbulence, eddy, density gradients, and shear forces influence both horizontal and vertical transport. Particle size, density, shape, surface chemistry, and organic coating properties determine settling rates and resuspension tendencies. During diffusion, the plume continuously mixes and exchanges with the surrounding water body, causing a concentration decrease. Boundary processes such as bottom settling, lateral overflow, and intralayer return also alter transport pathways. Field monitoring indicates a rapid concentration decrease with distance: In mine tests, plume heights remained below 2 m within < 100 m of the source, while suspended solids concentrations remained significantly above background levels at 350 m. Primary sedimentation occurs within hundreds of meters, though trace suspended particles remain detectable at 4–5 km and, thus, may have some impact on the surrounding marine ecosystem [4–6].

Suspended particles begin settling under gravity or reduced turbulence and form redeposited layers, which cover benthic organisms and may alter seafloor structure. Small quantities of fine particles can be transported further through the mesopelagic layer, where they may be resuspended or enter large‐scale circulation. Thereby, they contribute to suspended background or parent material background concentrations [3, 7]. Process‐wise, the sequence “suspended solid diffusion → water column plume → settling and release” reflects sediment transport and distribution characteristics from disturbance sources to the environment. Sediment plume impacts on deep‐sea ecosystems can be analyzed across individual–community–system functional levels and temporal scales. Settling particles can bury benthic animals, filter feeders, corals, and sponges, thus causing suffocation effects, obstruction of respiratory/feeding passages, and impaired mobility [8]. Large particles or high‐velocity impacts may inflict mechanical damage to fragile tissues. High concentrations of suspended sediments reduce light transmission; although light plays a role in the deep sea, it may still affect photosynthetic organisms in shallow upwelling plume zones. Respiratory or feeding organs of filter feeders and suspension‐feeding predators may become clogged by particles, inducing chronic stress. Long‐term exposure to low sediment concentrations may inhibit growth, reproduction, or delay recovery [9, 10]. Mining operations may release heavy metals and trace elements (e.g., Cu, Ni, Co, Pb, and Zn) into the water column, which will cause sublethal or chronic toxic effects [11]. Local chemical conditions (pH, dissolved oxygen, nutrient salts, organic carbon, and organic matter concentrations) will become disturbed, too. Sediment particles rich in nutrients or organic matter may alter the micronutrient cycling of the benthic environment. This will induce oxygen consumption, bottom hypoxia, and sulfidation, thus creating adverse conditions [12, 13]. Disturbance as a consequence reduces benthic animal density and diversity and reshapes community composition. Even in recovery, species assemblages often differ from predisturbance states [14, 15].

Deep‐sea systems exhibit extremely long recovery cycles because several large benthic groups struggle to return to predisturbance levels for extended periods [16]. Pioneer species like xenophyophores may colonize disturbed areas, but the overall macrofauna remains sparse [17]. Loss of benthic functions (biological disturbance, organic matter decomposition, and nutrient cycling) impacts sediment–water interface coupling and ecosystem stability. Midwater plumes propagating tens of kilometers (backwash plumes) can affect filter‐feeding zooplankton and mesopelagic communities [18]. Zooplankton exposed to plume‐induced muddy conditions may exhibit tissue stress, increased mucus production, and gene expression of damage signals [24]. Midwater disturbances can propagate through food webs with cumulative effects and long‐term operations across multiple mining sites, which are potentially creating additive disturbances amplifying ecological impacts. Mining may cause changes in landforms and substrate structures, loss of hard substrates, and permanent destruction of biological habitats [25]. This will lead to the destruction of populations or communities dependent on specific ecological niches, resulting in irreversible ecological damage.

Several experiments provide empirical support (Table 4). CCFZ (1991–1993) conducted 49 disturbances across a 150 × 3000 m area. Short‐term and 9‐month monitoring revealed decreased small benthic invertebrate abundance and increased large benthic invertebrates, potentially due to enhanced food supply [26]. Japan Metal Mining Agency CCFZ (1994–1997) disturbed sence19 sections along two parallel 1600‐m tracks. Long‐term monitoring up to 2 years after disturbance showed that the small abundance of benthos recovered but with distinct species composition differences. Large‐sized groups did not reach predisturbance levels [27]. The DISCOL/Peruvian Basin (1988–1998) project consists of a 10.8 km^2^ area. Monitoring from 6 months to 7 years indicated long‐term community structure differences compared to undisturbed areas [29]. The 2025 OMCO 1979 track survey showed early colonizing organisms but sparse large‐sized colonizing animals, with track morphology still identifiable [17]. In deep‐sea mining tests, the primary sedimentation occurred within hundreds of meters of the source with faint deviations detectable at 4.5 km [6].

**Table 4:** Monitoring results of deep‐sea mineral exploitation on occurrence environment disturbance [113–116].

Researcher	Study area	Disturbance method	Duration of monitoring	Monitoring results
National Oceanic and Atmospheric Administration	Clarion–Clipperton fracture zone (CCFZ)	Deep‐sea sediment resuspension system	9 months	Abundance of some small benthic animals has declined; the number of large benthic animals has increased
Japan Metal Mining Agency	CCFZ	Deep‐sea impact testing using a deep‐sea sediment resuspension system	2 years	Following the conclusion of the experiment, the abundance of small benthic organisms in the sediment zone declined immediately; 2 years later, it recovered to its original level, though the species composition remained inconsistent. Furthermore, the abundance of certain large and macrobenthic groups remained lower than in undisturbed areas. After 17–18 years, the chemical composition of the sediments gradually approached its original state
University of Hamburg	Peruvian Basin of the Pacific	Disturbance and reattachment experiment: scraping the seabed	26 years later, monitor again	Certain benthic species populations may experience numerical recovery; the composition of recovered populations differs from that of undisturbed populations
Jones et al.	Simulated mining	Simulation of multimetal nodule mining disturbance	Estimated 20 years	Few animal populations recovered to baseline or control conditions after 20 years

Deep‐sea systems recover slowly and rarely return to baseline conditions. Current monitoring primarily involves small‐to‐medium‐scale, short‐term experiments, which lack large‐scale, long‐term data under real operational conditions. Current research indicates potential disturbance thresholds and irreversible risks [117, 118]. Future research directions should include establishing high‐resolution fluid–particle coupling models to predict plume evolution, conducting large‐scale, long‐term field monitoring, delineating ecologically sensitive zones and buffer areas, developing mitigation measures such as optimized mining methods and tailings treatment, and integrating theoretical and observational data for risk assessment and threshold studies.

## 5. Conclusion

Deep‐sea mineral resources are widely distributed and, as land‐based resources become difficult to exploit or their utilization rate decreases, more and more attention is being turned to the development and utilization of deep‐sea mineral resources. Deep‐sea mineral resources mainly include PMNs, Mn–iron crusts, and SMS deposits. These different types of deposits vary significantly in multiple aspects, such as their physical and chemical properties, formation mechanisms, metal content, geographical distribution, extraction technologies, and the environmental and social impacts associated with their exploitation. Despite these differences, all of them are rich in important metal mineral resources, including base metals (Cu, Co, and nickel), precious metals (gold and silver), and rare metals and REEs. The benthic impacts of deep‐sea mining mainly include sediment redeposition and heavy metal toxicity. The mining disturbance affects bottom turbidity, density, nutrients, and dissolved oxygen. Also, the operation of deep‐sea mining trucks can have a significant impact. As mining proceeds, the most significant issues are ecological restoration and tailing disposal. Deep‐sea mining will inevitably cause long‐term damage to ecosystems. Thus, comprehensive long‐term monitoring and assessment will be needed throughout the mining process to reduce the impacts and cost of ecological restoration. Not only mining but also the disposal of tailings can cause environmental problems. Consequently, it is crucial to increase environmentally adaptive management systems and adopt innovative approaches to tailing disposal. With the development of modern biotechnology, bioleaching is expected to make breakthroughs. Genetic engineering can be adopted to modify microorganisms, which can improve leaching efficiency. Therefore, innovative technologies need to pay more attention to ecological environmental protection and the sustainable use of resources in order to develop efficient and environmentally friendly processes.

## Author Contributions

Jia Liu was responsible for investigation, original draft, and data curation; Can Wang was responsible for conceptualization, original draft, and data curation; Ini‐Ibehe Nabuk Etim was responsible for editing and supervision; Ruiyong Zhang was responsible for funding acquisition, editing, review, and supervision; Wolfgang Sand was responsible for editing, supervision, conceptualization, and review; Luhua Yang was responsible for editing and formal analysis; Xiao Wang was responsible for supervision; Yanchen Ge was responsible for editing; Jiazhi Liu was responsible for conceptualization. Jia Liu and Can Wang contributed equal work to this paper.

## Funding

The study was funded by the National Key Research and Development Program of China (grant No. 2024YFF0510100) and Institute of Oceanology, Chinese Academy of Sciences (grant No. E32832101H).

## Conflicts of Interest

The authors declare no conflicts of interest.

## Data Availability

The data that support the findings of this study are available from the corresponding author upon reasonable request.
